# The Caregiving Burden of Older People with Functional Deficits and Associated Factors on Malaysian Family Caregivers

**DOI:** 10.21315/mjms2024.31.1.14

**Published:** 2024-02-28

**Authors:** Patimah Abdul Wahab, Nurul Afiah Abdul Talib, Nik Noor Kaussar Nik Mohd Hatta, Sanisah Saidi, Zamzaliza Abdul Mulud, Muhammad Nubli Abdul Wahab, Hasanah Pairoh

**Affiliations:** 1 Department of Medical Surgical Nursing, Kulliyyah of Nursing, International Islamic University Malaysia Kuantan Campus, Pahang, Malaysia; 2 Department of Operation Theatre, Gleneagles Hospital Medini, Johor, Malaysia; 3 Centre for Nursing Studies, Faculty of Health Sciences, Universiti Teknologi MARA, Selangor, Malaysia; 4 Centre of Human Science, Universiti Malaysia Pahang, Pahang, Malaysia; 5 Faculty of Nursing, Prince of Songkhla University, Pattani Campus, Pattani, Thailand

**Keywords:** aged, caregiver burden, family caregivers, health status

## Abstract

**Background:**

Providing care to older people can be an extremely complex task, given their increased functional deficits, which may lead to family caregivers experiencing burnout and a deteriorated health status. This study investigated the caregiving burden of older people with functional deficits on family caregivers and associated factors.

**Methods:**

This cross-sectional study was conducted on family caregivers of older people with functional deficits living in FELDA schemes in Pahang, Malaysia. A self-administered questionnaire was used to collect data, which included the sociodemographical profile, health status and caregiving demands factors. The caregiving burden was assessed using the Malay version of the Zarit Burden Interview (ZBI). Multiple linear regression was used to assess the factors associated with burden.

**Results:**

A total of 271 family caregivers completed the questionnaire. Their mean age was 45.8 (SD 0.9) years old. The mean score for caregiving burden was 18.5 (SD 13.6). Caregivers’ gender (3.5 [95% CI: 0.2, 6.8]; *P =* 0.037), older people with chronic disease (9.6 [95% CI: 2.4, 16.9]; *P =* 0.010) and the functional independence of older people (−1.1 [95% CI: −1.6, −0.6]; *P <* 0.001) were predictors of family caregiving burden.

**Conclusion:**

The caregiving burden among family caregivers was mild and influenced mainly by care recipients’ health status. A proper assessment should be conducted and relevant health education provided to prepare family caregivers to care for their family members before discharge from the hospital.

## Introduction

The ageing population is increasing, with the global population of those aged 60 years and above estimated to double by 2050 (1). In 2020, 6.8% of the Malaysian population was older people aged 65 years old and above, compared to 5% in 2010 (2). Worldwide, care for older people at the community level generally occurs at home and is given by family caregivers (3). They perform caregiving by providing direct physical and emotional support, typically unpaid, to older people or family members who have become dependent or need some care or assistance (4, 5). Family caregiving is informal, and care is provided at various levels of competency, skill and motivation to assist (5).

Functional deficits in older people are a global issue and are associated with an increase in dependency on family caregivers. Caregiving activities, which become more challenging when needs become increasingly complex and demanding, involve activities such as providing basic hygienic care, a conducive care environment and a well-balanced diet. Furthermore, care providers must act as companions during hospital visits. The consequences of an overload of caregiving activities on family caregivers include a high burden and psychological distress, which can lead to a deterioration in health status and a poor quality of life (5, 6). In more extreme cases, stress, anxiety and depression were found to cause crises due to increased suicidal ideation among family caregivers (7, 8). Among ageing spousal caregivers, caregiving usually results in an increased risk of frailty, shorter nights of sleep and difficulties maintaining their social network (9, 10).

The factors influencing caregiving burden are widely acknowledged to be complex and multidimensional. They include demands and resources, caregiver setting and social environment (11). The experience of burden is subjective because family caregivers are affected differently, depending on the care demands and their experiences throughout the disease process. Specific factors that are relatively robust predictors of negative psychological effects are caregiving intensity, being female, being the wife of the care recipient, living with the care recipient and challenging behavioural symptoms in the care recipient (5). The ageing process and chronic diseases, such as stroke, dementia and Alzheimer’s, are common problems contributing to older people’s poor functional status, thus increasing the intensity of caregiving. An increase in older people’s functional deficits and their dependency on caregivers to complete activities of daily living (ADL) were among the most potent predictors of caregiving–work conflict (12), which may result in substandard or poor care and an increased risk of neglect and mortality in older people (13, 14).

Existing studies in Malaysia have focused on caregiving for individuals with specific diseases or conditions, including mental illness, palliative care, Alzheimer’s and epilepsy (15–18). Most findings have shown that Malaysian family caregivers experience mild to moderate burden (15, 16, 18). In Malaysia, the factors of being socially assigned, morally obliged and intrinsically assumed to have to care for an unwell family member often lead children to take care of their parents (16). When their spouse has passed on, older people often live with their children, who then provide care (19). However, massive urban migration in Malaysia, especially inter-urban and rural-urban migration among young adults in search of better life opportunities, represents a challenge in their ability to care for older people. In 2020, ‘family’ continued to be reported as the main reason for migration, with about 45.3% of respondents stating this, followed by ‘career’ (23.6%) and ‘the environment’ (22.3%) (20). Therefore, this study aims to, first, investigate the caregiving burden caused by older people with functional deficits and, second, determine the factors associated with this burden on family caregivers living in Federal Land Development Authority (FELDA) schemes in Pahang.

## Methods

A cross-sectional design was used to assess participants recruited from FELDA schemes in Pahang, Malaysia. The study was conducted between April 2021 and February 2022. FELDA is one of the rural transformation agencies established by the Malaysian government to overcome the high rural poverty rate. It has been 66 years since FELDA’s development. As such, most early generations of FELDA settlers are already in very late adulthood and almost half were unable to perform at least one of the instrumental ADLs (21). The study participants comprised the family caregivers of older people with functional deficits. The inclusion criteria were Malaysians aged 18 years old and above who had lived in FELDA schemes for a minimum of 12 months, were a primary family caregiver to older people with functional deficits and were able to understand and communicate in English or Malay. People were excluded if they had significant sensory disabilities or hearing problems or if they were mute, deaf or illiterate.

The care recipients were older people with functional deficits defined as being aged 60 years old or above during data collection. The Malay version of the Barthel Index (22) was used to screen for functional independence in older people. The score ranged between 0 and 20, where a low score indicated high dependence on others to complete their ADL and a high score indicated independence in performing ADL. A score between 0 and 19 indicated that the older person had functional deficits. The primary family caregiver was defined as the person most involved in taking care of older people at home and was mainly determined by the older people interviewed.

Random cluster sampling was used in participant recruitment. The family caregivers were clustered by the FELDA schemes in which they lived, resulting in four schemes out of 42 being selected: i) FELDA Sungai Panching Timur, ii) FELDA Bukit Goh, iii) FELDA Bukit Sagu 01 and iv) FELDA Bukit Sagu 02/03. Before the recruitment of the participants, older people from the selected FELDA schemes were identified and screened for functional deficit status. If they were indicated as having functional deficits and the eligibility of their family caregivers was confirmed, they were invited to participate in the study. The authors identified and recruited the participants primarily through a door-to-door survey assisted by each respective FELDA scheme Development Committee. The estimated sample size was calculated using PS Power and Sample Size Calculations software version 3.1.6 (23) based on a standard deviation of 14 (16), an alpha value of 0.05 and a power of 0.80, while the true difference of means was set at 5. After incorporating a 20% dropout rate, the sample size required was determined to be 298.

### Data Collection

To collect the data, a self-administered questionnaire was individually hand delivered to each participant. Verbal instructions were provided to the participants who were asked to complete the questionnaire by following the instructions for each part. Their responses involved either ticking or circling the best answer that represented their condition. Each questionnaire was collected three days later. A telephone interview was conducted with several participants who did not complete the questionnaire. The questionnaire was designed to obtain information about participants’ socio-demographic profile, health status, caregiving demands and caregiving burden.

The sociodemographic profile captured each participant’s age, gender, race, marital status, education level, employment status, monthly income, number of people in the household and role in the family. The portion on their self-reported health status was used to obtain data on the participants’ underlying health problems, smoking status (yes/no), alcohol intake (yes/no), body weight and height. In the ‘smoking status’ section, they were asked to state the frequency at which they smoked daily if they were smokers. Body mass index (BMI) was calculated based on self-reported body weight and height using the following formula: body weight (kg)/(height [m] × height [m]).

To enable the assessment of the demands of caregiving, the participants were asked to provide data on the sociodemographic background of the older people (care recipients), their own health problems, their estimated hours per day spent taking care of older people and the number of older people they were taking care of at the time. The Malay version of the Zarit Burden Interview (ZBI) was used to measure caregiving burden, which has been demonstrated to be valid and reliable for use with the Malaysian population (24). The Cronbach’s alpha of the ZBI in the current study was 0.91. The scale comprised 22 items, which were scored using 5-point Likert scales ranging from 0 (never) to 4 (nearly always). The item scores were summed to give the total score, which ranged from 0 to 88. A high score indicated a greater caregiving burden.

### Statistical Analysis

The data were analysed using the IBM Statistical Package for Social Science (SPSS) for Windows, version 26.0. Descriptive statistical methods to calculate frequency and percentage were used to present the categorical data, and the mean (SD) was used to present the numerical data (the background of the participants and the family caregiving burden scores). Simple linear regression was used to assess the factors associated with family caregiving burdens. Independent variables with *P*-values < 0.25 were included in the multiple linear regression. Forward, backward and stepwise methods were applied to select variables in the model. The two-way interaction between the independent variables was checked and a *P-*value > 0.05 indicated there was no interaction between them. Multicollinearity was checked using the variance inflation factor (VIF). A VIF value of less than 10 indicated that there was no multicollinearity problem among the variables. A *P*-value ≤ 0.05 was set as the level of significance.

## Results

### Background of the Participants

A total of 271 participants took part in this study. Their mean age was 45.8 (SD 0.9) years old, and all were Malay and Muslim. More than half were female (66.4%) and the majority were employed (59.0%). Most participants were married (73.4%), had received secondary- and tertiary-level education (75.6%) and had lived in a nuclear family structure (76.0%). About 52.8% had some health problems and 13.3% were smokers who smoked an average of 0.9 (SD 3) cigarettes per day. The average BMI of the participants was 26.4 (SD 4.4) kg/m^2^. The characteristics of the study participants are presented in Table 1.

### Caregiving Burden and Demands

The mean score of the caregiving burden was 18.5 (SD 13.6), with a range between 0 and 76. The care recipients mean functional independence score was 17.7 (SD 3.2). The average number of hours per day participants spent providing care to older people with functional deficits was 14.4 (SD 6.8). Some participants cared for more than one older person (mean = 1.2, SD 0.4).

The mean age of the care recipients was 69.5 (SD 6.3) years old. Among the 271 care recipients, 54.2% were women and 45.8% were men. About 59.4% were married and 40.6% were single. Most care recipients had received primary education, had never been to school or attended informal school (84.1%). Most were unemployed or retired (71.6%) and had a personal income of less than RM2,000 (72.7%). Almost all care recipients had chronic diseases, particularly hypertension, diabetes mellitus type 2 and hyperlipidaemia (95.2%). Table 2 shows the characteristics of the care recipients and the caregiving demands linked to caring for older people with functional deficits.

### Associated Factors

Table 3 summarises the univariable and multivariable analyses of the caregiving burden predictors. Among the family caregivers’ factors, gender (*P* = 0.023) and personal monthly income (*P* = 0.034) were significantly associated with a high burden from providing care for older people with functional deficits, as demonstrated by the univariable analysis. Among the care recipient factors, the presence of chronic disease (*P* = 0.006) was found to be significantly associated with a high caregiving burden. Meanwhile, among the factors linked to caregiving demands, only the functional independence of older people (*P* < 0.001) was significantly associated with family caregiving burden.

In the multivariable analysis, independent significant factors for family caregiving burden were found to be the family caregiver’s gender (3.5 [95% CI: 0.2, 6.8]; *P* = 0.037), the presence of chronic disease in older people with functional deficits (9.6 [95% CI: 2.4, 16.9]; *P* = 0.010) and the functional independence of older people with functional deficits (−1.1 [95% CI: −1.6, −0.6]; *P* < 0.001). Based on the multiple linear regression model *(R*^2^ = 0.115), these variables together explained 11.5% of the variance in caregiving burden among family caregivers.

## Discussion

This study investigated the caregiving burden of caring for older people with functional deficits, as well as associated factors, on family caregivers living in FELDA schemes in Pahang, Malaysia. The findings illustrated that family caregivers experienced a mild burden when taking care of older people with functional deficits, which was not greatly dissimilar to the worldwide findings (25, 26). These findings are also comparable to those from previous Malaysian studies on the family caregivers of adults with epilepsy, individuals with severe mental illness and palliative care patients; specifically, the findings showed that the caregiving burden was reported to be mild to moderate (15, 16, 18). Of the existing studies, two used the same instrument (the ZBI) as in this study to measure caregiving burden (16, 18).

In comparison to the findings of Ahmad Zubaidi et al. (16) and Lai et al. (18) (mean = 23.33 [SD 13.70] and mean = 29.93 [SD 16.90], respectively), the current findings showed the lowest mean score (mean = 18.49, SD 13.64), indicating that our cohort of participants had a less negative experience providing care to family members. A survival analysis of older people and their family caregivers in the US found that the mortality risk among older people whose caregivers perceived no burden was lower than the risk among those whose caregivers reported a burden (14). Despite the heterogeneity in the reporting of caregiver burden measures evident in studies worldwide, concrete findings have shown that the family caregivers of older people tend to experience a caregiving burden, including anxiety and depression, particularly when they have to care for physically frail older people (6, 27).

The provision of care might have a greatly varied impact, largely determined by the degree of care given and the suffering of the recipient (5). Michel et al. (6) conducted a study among family caregivers in Western countries and discovered that differences in health and social care systems were among the factors that influenced their findings. Meanwhile, social support was found to have a fully mediating effect on the relationship between resilience and burden (25). Regarding the current study, the participants were community-dwelling family caregivers living in land development projects. They engaged in agricultural activities and were members of an industrial and commercial social economy. Most were self-employed, worked on their farms and lived with or near older people. The community areas were fully equipped with healthcare facilities and the residents’ welfare was managed by each scheme’s development committee. Therefore, it is postulated that being able to obtain social support from the surroundings and community resources probably influenced the general perception that the caregiving role involved a mild burden.

In this study, we found that women were associated with a caregiving burden four times greater than that experienced by men. Furthermore, women comprised two-thirds of the family caregivers. This finding is consistent with those of previous studies that detected a higher level of caregiving burden among women than men (18, 28). Women were found to perform caregiving duties as though they were home chores without complaining, provide extensive assistance more often than men and frequently underestimate their need for support (14, 18, 29). Women were also found to frequently cope with the stress of caregiving by laughing off the consequences, emphasising the virtues of selflessness and by repressing their feelings and losing interest in their duties (29). In contrast, Gérain and Zech (11) showed that the caregiver’s age had no stable effect on the perceived burden level, suggesting that this was due to the different implications of the caregiving role at different life stages.

The current findings also indicate that compared to when care recipients exhibited no symptoms of chronic disease, the presence of such disease among this group was 10 times more likely to result in a caregiving burden among family caregivers. Most care recipients in this study suffered from chronic diseases, particularly hypertension, diabetes mellitus type 2 and hyperlipidaemia, which could be attributed to the possibility that chronic disease accelerates the development of functional deficits in older people. A study exploring the association between chronic conditions and ADL limitations among older people in India found that those with pre-existing chronic conditions had a higher likelihood of low competency in completing ADL (30). The chance of having a low ability to complete ADL was two times higher among older adults with more than three chronic conditions (30). Conversely, aside from the number of chronic diseases, the caregiving burden may differ depending on the type of disease, diagnosis, treatment and disease progression (31). For example, previous studies have shown that of the common diseases in older people, dementia is frequently associated with a caregiving burden (8, 32).

Mental and physical health impairments due to disease progression subsequently lead to greater functional deficits, thus increasing older adults’ level of dependency on family caregivers (17, 33). The current findings support those of previous studies in that high functional independence among older people was an independent significant factor linked to a low caregiving burden among family caregivers (17, 26, 34, 35). Overall, most care recipients in this study were rated as having high functional independence. The care recipients’ low demand for assistance in performing ADL supports the discovery of a mild caregiving burden among the participants. Other factors that may have influenced the present findings, such as self-efficacy, and cultural and spiritual factors should be considered in future studies.

One limitation of this study is that it relied on self-reported measures, which may have led to risk recall bias. The study sample was limited to family caregivers from FELDA schemes in Pahang, so the findings cannot be generalised to urban areas. The study was conducted during the COVID-19 pandemic, which influenced the study methods in terms of gaining access to the participants due to the movement control order enforced by the government to break the national COVID-19 infection chain. In addition, the researchers did not look specifically at older people who had moved to urban areas to be taken care of by their children, possibly due to a higher functional deficit or the caregiving burdens resulting from severe caregiving-work conflict (36). Despite these limitations, substantial associations were observed in several outcome measures. To the authors’ knowledge, this study is the first to assess the caregiving burden caused by older people with functional deficits living in the largest set of land development projects in Malaysia (that is, FELDA).

## Conclusion

The key findings of this study showed that the family caregivers of older people with functional deficits living in FELDA schemes in Pahang experienced a significant burden due to their caregiving tasks. Women caregivers and high caregiving demands due to care recipients’ poor health status and functional dependence were found to be primarily associated with an increased burden. Among these, the presence of chronic diseases in older people is the highest predictor of burden. Future research is required to study the caregiving burden in urban areas, as well as to consider other possible factors, such as ethnicity, social support, spiritual factors and the rewarding role of caregiving.

## Figures and Tables

**Table 1:** Characteristics of the family caregivers (*n* = 271)

Variables		*n* (%)	Mean (SD)
Age (years old)			45.8 (0.9)
Gender	Male	91 (33.6)	
	Female	180 (66.4)	
Marital status	Married	199 (73.4)	
	Single/divorce/widowed	72 (26.6)	
Education level	Never/primary	66 (24.4)	
	Secondary/tertiary	205 (75.6)	
Employment status	Unemployed/retired	111 (41.0)	
	Employed	160 (59.0)	
Personal income (RM)	< 1,000	79 (29.2)	
	1,000 or above	192 (70.8)	
Household income (RM)	< 2,000	168 (62.0)	
	2,000 or above	103 (38.0)	
Number of households			5.8 (2.4)
Types of family structure	Nuclear	206 (76.0)	
	Extended	65 (24.0)	
Head of the family	Yes	93 (34.3)	
	No	178 (65.7)	
Smoking status	Yes	36 (13.3)	
	No	235 (86.7)	
BMI (kg/m^2^)			26.4 (4.4)
Health problems	Yes	143 (52.8)	
	No	128 (47.2)	

Notes: BMI = body mass index; RM = Malaysian Ringgit; SD = standard deviation

**Table 2:** Characteristics of the care recipients (*n* = 271) and the caregiving demands

Variables		*n* (%)	Mean (SD)
Age (years old)			69.5 (6.3)
Gender	Men	124 (45.8)	
	Women	147 (54.2)	
Marital status	Married	161 (59.4)	
	Single/divorce/widowed	110 (40.6)	
Educational level	Never/informal school	68 (25.1)	
	Primary	160 (59.0)	
	Secondary/tertiary	43 (15.9)	
Employment status	Unemployed/retired	194 (71.6)	
	Employed	77 (28.4)	
Personal monthly income (RM)	< 1,000	83 (30.6)	
	1,000–1,999	114 (42.1)	
	2,000 or above	74 (27.3)	
Smoking status	Yes	48 (17.7)	
	No	223 (82.3)	
BMI (kg/m2)			25.8 (4.0)
Presence of any chronic disease	Yes	258 (95.2)	
	No	13 (4.8)	
Functional independence			17.7 (3.2)
Hours spent for caregiving per day			14.4 (6.8)
Number of older people as the care recipient			1.2 (0.4)

Notes: BMI = body mass index; RM = Malaysian Ringgit; SD = standard deviation

**Table 3:** Factors associated with family caregiving burden (*n* = 271)

Factors		Crude *b* (95% CI)^a^	*P-*value	Adjusted *b* (95% CI)^b^	*P-*value
***Family caregivers***					
Age (years old)		0.1 (−0.0, 0.2)	0.141	–	–
Gender	Women versus Men^c^	4.0 (0.6, 7.4)	0.023	3.5 (0.2, 6.8)	0.037
Employment status	Employed versus Unemployed^c^	−3.1 (−6.4, 0.2)	0.064	–	–
Personal monthly income (RM)	1,000 or above versus < 1,000^c^	−3.9 (−7.4, −0.3)	0.034	–	–
Types of family structure	Extended versus Nuclear^c^	−3.3 (−7.1, 0.5)	0.091	–	–
Head of the family	Yes versus No^c^	−3.2 (−6.6, 0.2)	0.065	–	–
BMI (kg/m^2^)		0.2 (−0.1, 0.6)	0.208	–	–
Presence of chronic disease	Yes versus No^c^	3.0 (−0.3, 6.2)	0.075	–	–
***Care recipients***					
Age (years old)		0.2 (−0.1, 0.5)	0.095	–	–
Gender	Women versus Men^c^	−2.8 (−6.0, 0.5)	0.096	–	–
BMI (kg/m2)		0.4 (−0.0, 0.8)	0.069	–	–
Presence of chronic disease		10.7 (3.2, 18.2)	0.006	9.6 (2.4, 16.9)	0.010
***Demands in caregiving***					
Functional independence		−1.2 (−1.7, −0.7)	< 0.001	−1.1 (−1.6, −0.6)	< 0.001
Time spent for caregiving (hours)		0.2 (−0.1, 0.4)	0.124	–	–

Notes: BMI = body mass index; CI = confidence interval; RM = Malaysian Ringgit;

a Crude regression coefficient;

b Adjusted regression coefficient;

c The reference category; Stepwise, forward and backward multiple linear regression method applied. Model assumptions were fulfilled. There were no interactions among independent variables. No multicollinearity was detected. Coefficient of determination (*R*^2^) = 0.115. A significant *P*-value was set at 0.05
